# Anesthetic Considerations in ESRD Secondary to Lupus Nephritis

**DOI:** 10.7759/cureus.105508

**Published:** 2026-03-19

**Authors:** Kiran Ghotra, Chloe Grabowski, Michael Weber

**Affiliations:** 1 Anesthesiology, Lake Erie College of Osteopathic Medicine, Trenton, USA; 2 Anesthesia, Michigan State University College of Osteopathic Medicine, Brownstown, USA; 3 Anesthesia, Corewell Health Trenton Hospital, Trenton, USA

**Keywords:** anesthesia management, end-stage renal disease, hemodialysis, lupus nephritis, perioperative care, sugammadex, systemic lupus erythematosus

## Abstract

Systemic lupus erythematosus (SLE) is a chronic autoimmune disease that can affect multiple organ systems, and kidney involvement in the form of lupus nephritis is common. In some patients, lupus nephritis can progress to end-stage renal disease (ESRD), requiring renal replacement therapy. Patients with ESRD secondary to lupus nephritis present unique challenges for anesthetic management due to impaired renal drug clearance, electrolyte disturbances, cardiovascular comorbidities, hematologic abnormalities, and the multisystem manifestations of SLE. We present the case of a 23-year-old woman with biopsy-proven lupus nephritis and ESRD on hemodialysis who underwent fitting and adjustment of a peritoneal dialysis catheter while awaiting kidney transplantation. Anesthetic management focused on maintaining hemodynamic stability while avoiding medications that may accumulate in the setting of renal failure. Induction of anesthesia was performed with propofol, fentanyl, and rocuronium, and anesthesia was maintained with sevoflurane. Neuromuscular blockade was reversed with sugammadex at the conclusion of the procedure. This case highlights the importance of careful medication selection and perioperative planning when providing anesthesia for patients with ESRD secondary to lupus nephritis.

## Introduction

Systemic lupus erythematosus (SLE) is a chronic autoimmune disease that can affect multiple organ systems, with the kidneys being one of the most commonly involved organs [1-8]. Lupus nephritis develops in approximately 40%-60% of patients with SLE, and about 10%-22% of these patients eventually progress to end-stage renal disease (ESRD) requiring dialysis or kidney transplantation [1-8]. Managing patients with ESRD in the perioperative setting can be challenging due to changes in drug metabolism, electrolyte abnormalities, anemia, platelet dysfunction, and increased cardiovascular risk [2-5]. Patients with SLE may also face additional perioperative risks compared to the general population [1]. Studies have shown higher rates of postoperative renal complications and increased in-hospital mortality after major surgery in this population [1]. Cardiovascular disease is the leading cause of death among patients with ESRD, making careful cardiovascular evaluation an important part of perioperative planning [4]. Additionally, lupus-related complications such as pericarditis, neurologic involvement, and hematologic abnormalities can further influence anesthetic management [7].

Medication selection is particularly important when administering anesthesia to patients with ESRD [2-5]. Because renal clearance is reduced, certain drugs may accumulate and lead to prolonged effects or toxicity [2-5]. As a result, many anesthetic agents require dose adjustments or should be avoided in patients with significant renal impairment [2-4]. Short-acting medications and drugs that rely primarily on hepatic metabolism or organ-independent elimination are generally preferred [2-4]. Careful anesthetic decision-making is therefore essential in patients with lupus nephritis progressing to ESRD, as perioperative physiologic stress and altered pharmacokinetics may increase the risk of complications. In this report, we describe the anesthetic management of a young patient with ESRD secondary to lupus nephritis who underwent laparoscopic peritoneal dialysis catheter placement while awaiting kidney transplantation.

## Case presentation

A 23-year-old African American woman with a body mass index of 16.82 kg/m² (underweight), classified as American Society of Anesthesiologists (ASA) Physical Status IV, with a history of systemic lupus erythematosus (SLE), presented for elective peritoneal dialysis catheter placement while awaiting kidney transplantation. She was initially diagnosed with SLE in November 2024. At the time of diagnosis, laboratory testing showed positive antinuclear antibodies (ANA), anti-double-stranded DNA antibodies, and SSA antibodies. Her disease manifestations included hypocomplementemia, anemia, thrombocytopenia, seizures, and Evans syndrome. She previously required treatment with intravenous immunoglobulin, romiplostim (N-plate), rituximab, and cyclophosphamide. Renal biopsy demonstrated immune complex-mediated glomerulonephritis with prominent mesangial and subendothelial electron-dense deposits, consistent with class III or IV lupus nephritis.

Her disease progressed to end-stage renal disease (ESRD) with nephrotic range proteinuria, and she began hemodialysis three times weekly one year and four months prior to surgery. Her additional medical history included hypertension, anemia, thrombosis, and a prior pericardial effusion with tamponade that required a pericardial window less than a year prior to surgery. Her home medications included carvedilol, nifedipine, hydralazine, atorvastatin, hydroxychloroquine, mycophenolate, lacosamide, and furosemide, along with folic acid and renal multivitamins.

Preoperative considerations

Patients with ESRD undergoing surgery require careful preoperative evaluation [4,5]. In this patient, laboratory studies focused on renal function, electrolyte abnormalities, anemia, and coagulation status (Table 1). The patient demonstrated leukopenia, anemia, and mild hypokalemia, consistent with her underlying chronic disease and renal dysfunction. Cardiovascular evaluation was also an important component of the preoperative assessment, as cardiovascular disease is common in patients with ESRD, and SLE may also involve the heart, including complications such as pericarditis [4-7]. Preoperative cardiac evaluation included an electrocardiogram and a transthoracic echocardiogram, which demonstrated a preserved ejection fraction of 76% with mild pulmonic and tricuspid regurgitation and no evidence of pericardial effusion. Cardiology clearance was obtained, and the patient was assessed as low cardiac risk (approximately 1%) for the planned peritoneal dialysis catheter placement. Patients with lupus nephritis may also be screened for antiphospholipid antibodies, as these antibodies increase the risk of thrombosis and may contribute to complications involving dialysis catheters [6-8]. In this case, the patient tested negative for these antibodies. During surgery, maintaining stable hemodynamics and adequate renal perfusion is important to reduce the risk of further renal injury or systemic complications [5]. Patients with SLE have also been shown to have higher rates of postoperative renal complications compared with the general population [1].

**Table 1:** Preoperative Hematologic, Biochemical, and Immunologic Laboratory Findings Laboratory values were obtained within 48 hours of the patient’s most recent hemodialysis session and are presented with patient results, units, and reference ranges. Values outside the normal range are indicated as “Low” or “High.” Hematologic, biochemical, and immunologic parameters were assessed as part of the preoperative evaluation for anesthesia planning. WBC: white blood cell count, HGB: hemoglobin, HCT: hematocrit, BUN: blood urea nitrogen, eGFR: estimated glomerular filtration rate, ANA: antinuclear antibodies

Laboratory test	Patient value	Units	Reference range
Hematology			
WBC	2.5 (Low)	×10³/µL	4-11
HGB	9.7 (Low)	g/dL	12-16
HCT	29.6 (Low)	%	36-46
Platelet count	201	×10³/µL	150-450
Chemistry			
Sodium	142	mEq/L	135-145
Potassium	3.4 (Low)	mEq/L	3.5-5.0
Chloride	99	mEq/L	98-107
BUN	15	mg/dL	7-20
Creatinine	2.70 (High)	mg/dL	0.6-1.3
eGFR	25 (Low)	mL/min/1.73 m²	>60
Calcium	9.5	mg/dL	8.6-10.2
Glucose	85	mg/dL	70-99
Hemoglobin A1c	5.3	%	<5.7
Immunologic/autoantibody testing			
ANA	Positive	-	Negative
Anti-double-stranded DNA antibodies	Positive	-	Negative
SSA antibodies	Positive	-	Negative

Anesthetic considerations and contraindications

In patients with ESRD, reduced renal clearance can lead to accumulation of medications and prolonged drug effects [2-5]. Because of this, anesthetic drug selection requires careful consideration [2-5]. Electrolyte abnormalities, particularly hyperkalemia, can also influence which medications are safe to use [2-5]. Several medications were avoided during this case. Succinylcholine was avoided because it can cause a transient increase in serum potassium of approximately 0.5-1 mEq/L, which may trigger arrhythmias in patients who are already at risk for hyperkalemia [2,3]. Pancuronium was also avoided because it is largely excreted by the kidneys and may result in prolonged neuromuscular blockade in patients with renal impairment [2,3]. Morphine was avoided due to the accumulation of active metabolites that are cleared renally and can lead to prolonged respiratory depression in patients with ESRD [2-4].

Neuromuscular blocking agents such as cisatracurium or atracurium are often preferred in patients with renal failure because they undergo Hofmann elimination rather than renal excretion [2,3]. In this case, however, rocuronium was used because sugammadex was available for rapid reversal if needed. In general, short-acting medications and drugs that rely primarily on hepatic metabolism are preferred in patients with ESRD to reduce the risk of drug accumulation [2-4].

Anesthetic plan and intraoperative management

Both regional and general anesthesia can be used in patients with ESRD, although medication selection must account for altered pharmacokinetics and renal clearance [2,3]. In this case, general anesthesia was selected based on the requirements of the procedure.

The patient received midazolam 2 mg for anxiolysis and fentanyl 100 mcg for analgesia. Lidocaine 2% was administered during induction to help suppress airway reflexes. Anesthesia was induced with propofol 200 mg, and neuromuscular blockade was achieved with rocuronium 40 mg. Dexamethasone 4 mg and ondansetron 4 mg were given for postoperative nausea and vomiting prophylaxis. Glycopyrrolate 0.2 mg was administered for its antisialagogue effects, and cefazolin 1 g was administered for perioperative antibiotic prophylaxis, consistent with weight-based surgical prophylaxis recommendations in patients weighing less than 80 kg. Diphenhydramine 12.5 mg was also administered as additional prophylaxis for potential allergic reactions.

Anesthesia was maintained with sevoflurane, which is commonly used in patients with renal dysfunction due to its favorable pharmacokinetic profile [2,3]. The surgical procedure lasted approximately 90 minutes. Throughout the procedure, the patient remained hemodynamically stable, with mean non-invasive blood pressure ranging from approximately 100-115 mmHg, systolic BP 100-115 mmHg, diastolic BP 50-75 mmHg, heart rate 65-70 bpm, and oxygen saturation consistently ≥99%. No clinically significant hypotension, hypertension, or arrhythmias were observed. Estimated blood loss was minimal (approximately 5 mL). Mechanical ventilation was performed using pressure-controlled ventilation with volume guarantee (PCV-VG). Fraction of inspired oxygen ranged from 65% to 99%, and end-tidal carbon dioxide was maintained between 33 and 43 mmHg throughout the procedure. At the conclusion of the procedure, neuromuscular blockade was reversed with sugammadex 200 mg. Sugammadex works by binding rocuronium and allowing rapid reversal of neuromuscular blockade. A total of 300 mL of normal saline was administered during the procedure to maintain hemodynamic stability while minimizing the risk of fluid overload.

Postoperative course

The patient tolerated the procedure without complications. Hemodynamic parameters remained stable throughout the operation, and no significant electrolyte abnormalities were observed. Neuromuscular blockade was successfully reversed with sugammadex, and the patient was extubated without difficulty. In the recovery unit, she remained hemodynamically stable with adequate respiratory function. Given the increased perioperative risks associated with both SLE and ESRD, continued multidisciplinary follow-up with nephrology and rheumatology was recommended [1-6]. Coordination of care between these specialties is important in patients with lupus nephritis and ESRD and has been associated with improved long-term outcomes [6].

## Discussion

This case highlights the complexity of anesthetic management in patients with end-stage renal disease (ESRD) secondary to lupus nephritis [2-8]. Patients with systemic lupus erythematosus (SLE) and ESRD are at increased perioperative risk due to chronic inflammation, immune dysregulation, and multisystem involvement. Complications reported in this population include cardiovascular events such as arrhythmias or myocardial injury, thrombotic events related to antiphospholipid antibodies or dialysis access, and perioperative deterioration in renal function or electrolyte disturbances [1-7]. These patients may also experience altered drug metabolism due to renal impairment, increasing the risk of medication accumulation and prolonged pharmacologic effects [2-5]. Consequently, careful anesthetic planning, vigilant hemodynamic monitoring, and thoughtful medication selection are essential, with preference often given to agents that undergo hepatic metabolism or organ-independent elimination to minimize prolonged drug activity [2-4].

Neuromuscular blockade also requires careful attention [2,3]. Medications such as pancuronium, which are mostly cleared by the kidneys, can lead to prolonged paralysis in patients with ESRD [2,3]. Atracurium and cisatracurium are often preferred because they break down on their own without relying on kidney function [2,3]. Rocuronium can still be used safely when sugammadex is available for rapid and reliable reversal, particularly when neuromuscular blockade is monitored intraoperatively. In this case, neuromuscular blockade was monitored intraoperatively using train-of-four monitoring to guide dosing and ensure adequate reversal prior to extubation. Electrolyte imbalances are another concern. Hyperkalemia is common in ESRD, so succinylcholine is risky because it temporarily increases potassium levels, which could trigger dangerous heart rhythms [2-4]. Avoiding it helps reduce that risk.

Patients with SLE also face higher rates of complications after surgery [1]. Studies show they are more likely to develop kidney issues and have higher postoperative mortality [1]. Careful perioperative monitoring and involvement of multiple specialties are essential [1-6]. In the long run, kidney transplantation is the preferred treatment for ESRD, as it lowers the risk of death, cardiovascular problems, infections, and lupus flares compared with long-term dialysis [6-9]. Outcomes for patients with lupus nephritis are similar to those with other kidney diseases, and recurrence in the transplanted kidney is uncommon [6-9].

## Conclusions

Patients with ESRD due to lupus nephritis present distinct challenges for anesthetic management, largely related to altered drug pharmacokinetics, electrolyte abnormalities, and the multisystem effects of SLE. Careful perioperative planning is important, including thoughtful medication selection, close hemodynamic monitoring, and attention to electrolyte balance to reduce the risk of complications. In particular, it is important to avoid medications that can accumulate in renal failure or raise potassium levels, use shorter-acting agents when possible, and ensure appropriate reversal of neuromuscular blockade. This case demonstrates how individualized anesthetic planning and coordination across teams can support safe perioperative care in patients with complex disease. Although this is a single case with an uncomplicated course, it highlights practical considerations that may be helpful when managing similar patients.
